# Progressive femoral cortical and cancellous bone density loss after uncemented tapered-design stem fixation: A 6-year follow-up

**DOI:** 10.3109/17453671003635843

**Published:** 2010-04-06

**Authors:** Lutz A Mueller, Tobias E Nowak, Lothar Haeberle, Lars P Mueller, Alexander Kress, Michael Voelk, David Pfander, Raimund Forst, Rainer Schmidt

**Affiliations:** ^1^Department of Orthopaedic Surgery, Friedrich Alexander University of Erlangen-NurembergErlangen; ^2^Department of Trauma Surgery, Johannes Gutenberg University, Mainz; ^3^Department of Medical Informatics, Biometry and Epidemiology, Friedrich Alexander University of Erlangen-NurembergErlangen; ^4^Department of Orthopaedic and Trauma Surgery, Hufeland Klinikum GmbH, MuehlhausenGermany

## Abstract

**Background** Aseptic implant loosening and periprosthetic bone loss are major problems after total hip arthroplasty (THA). We present an in vivo method of computed tomography (CT) assisted osteodensitometry after THA that differentiates between cortical and cancellous bone density (BD) and area around the femoral component.

**Method** Cortical and cancellous periprosthetic femoral BD (mg CaHA/mL), area (mm^2^) and contact area between the prothesis and cortical bone were determined prospectively in 31 patients 10 days, 1 year, and 6 years after uncemented THA (mean age at implantation: 55 years) using CT-osteodensitometry.

**Results** 6 years postoperatively, cancellous BD had decreased by as much as 41% and cortical BD by up to 27% at the metaphyseal portion of the femur; this decrease was progressive between the 1-year and 6-year examinations. Mild cortical hypertrophy was observed along the entire length of the diaphysis. No statistically significant changes in cortical BD were observed along the diaphysis of the stem.

**Interpretation** Periprosthetic CT-assisted osteodensitometry has the technical ability to discriminate between cortical and cancellous bone structures with respect to strain-adapted remodeling. Continuous loss of cortical and cancellous BD at the femoral metaphysis, a homeostatic cortical strain configuration, and mild cortical hypertrophy along the diaphysis suggest a diaphyseal fixation of the implanted stem. CT-assisted osteodensitometry has the potential to become an effective instrument for quality control in THA by means of in vivo determination of periprosthetic BD, which may be a causal factor in implant loosening after THA.

## Introduction

Periprosthetic bone loss remains an important issue after THA. The degree of bone mineralization is a decisive parameter of bone quality (Mazess 1982, Kobayashi et al. 2000) and is correlated to bone stability (Houde et al. 1995). Studies from the Norwegian national hip register suggest that regional bone loss may contribute to the risk of aseptic loosening (Espehaug et al. 1997, Furnes et al. 2001). Furthermore, it may predispose to prosthetic migration, periprosthetic fractures, and to problems in revision arthroplasty (Willert et al. 1990). Thus, analysis of periprosthetic BD after THA is of some interest.

Cortical and cancellous bone structures have different modulus of elasticity and different mechanical characteristics, which are reflected by problems in the development of isoelastic implants (Draenert et al. 2005). These structural differences have not been taken into consideration when measuring periprosthetic BD after THA in vivo with DXA (Venesmaa et al. 2001, Brodner et al. 2004). However, using sectional views generated by computed tomography (CT), a separate analysis of cortical and cancellous bone structures can be achieved (Schmidt et al. 2005, Mueller et al. 2006).

Cementless prostheses have an intimate contact of up to 90% of their circumference with the surrounding cancellous bone over the proximal third of the implant (Schimmed and Huiskes 1988). Even so, to date only a limited number of studies have focused on the distribution of mechanical stress within cancellous bone. Finite element studies have indicated that poor quality of cancellous bone can promote subsidence of the prosthesis (Taylor et al. 1995a, b). Comparison of these results (Taylor et al. 1995a, b) with clinical migration data (Freeman and Plante-Bordeneuve 1994) has shown a high correlation and suggests that initial changes in cancellous BD may be predictive of implant migration and loosening (Freeman and Plante-Bordeneuve 1994, Taylor and Tanner 1997).

The objective of this study was to perform a prospective analysis of cortical and cancellous stress-related bone remodeling; cortical and cancellous area and contact area between the prothesis and the inner cortical bone after insertion of an uncemented stem using CT-assisted osteodensitometry in vivo. In addition, our aim was to evaluate the reproducibility of automated assessment of periprosthetic cortical and cancellous bone density.

## Patients and methods

Between 1998 and 2000, 31 consecutive primary uncemented total hip replacements (Cerafit Triradius-M press-fit cup and tapered-wedge Multicone stem; Ceraver Osteal, Paris, France) were performed in 31 patients with primary osteoarthritis (average age: 55 (36–75) years at index operation; 16 females). At the 6-year follow-up, 26 patients remained (2 patients had died, 2 suffered malignant disease, and 1 patient had moved overseas). The analysis is based on the remaining patients.

The inclusion criteria for uncemented total hip replacement were age less than 70 years, high functional demand, and bone quality type A or B according to the classification of Dorr et al. (1993). The exclusion criteria were rheumatoid arthritis, poor bone quality, bone deformity, fractures, systemic disease with marked impairment of function, and intake of bone-inducing drugs. All operations were performed by one experienced hip surgeon using a direct lateral approach. Postoperatively, touch weight bearing of up to 15 kg was allowed over the first 6 weeks, then progressively increased loading to full weight bearing within the next 2 weeks.

### Clinical and plain radiography measurements

Patients were assessed clinically using the Harris hip score. Radiographs were evaluated by an observer who was unaware of the clinical outcome. Evaluation of the femoral component was performed using published criteria (Johnston et al. 1990). Radiographic “stability” of the femoral component was assessed according to the criteria of Engh et al. (1987).

### CT measurements

To determine the precision of our measurements, 5 CT scans of 5 cadavers with uncemented total hip arthroplasty, using the same stem, were taken. The cadavers were repositioned after each measurement. The precision was calculated for cortical and cancellous BD; contact area; cortical and cancellous area. It was expressed as the coefficient of variation (CV) as a percentage according to the formula:


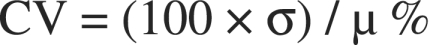


where µ is the overall mean of all the measurements and σ^2^ represents the within-subject variability. The values of σ and µ are estimated with the mixed linear model


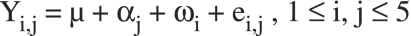


where Y_i,j_ is the j-th measurement of the i-th subject, α_j_ represents the fixed effect “time” and ω_i_ ∼ N (0, σ^2^_b_) and e_i,j_ ∼ N (0, σ^2^) are random variables. This calculation generalizes the well-established CV for paired measurements and thus allows more than 2 repeated measurements. For 2 instead of 5 measurements, the CV as described above coincides with the well-established CV.

**Figure 1.**
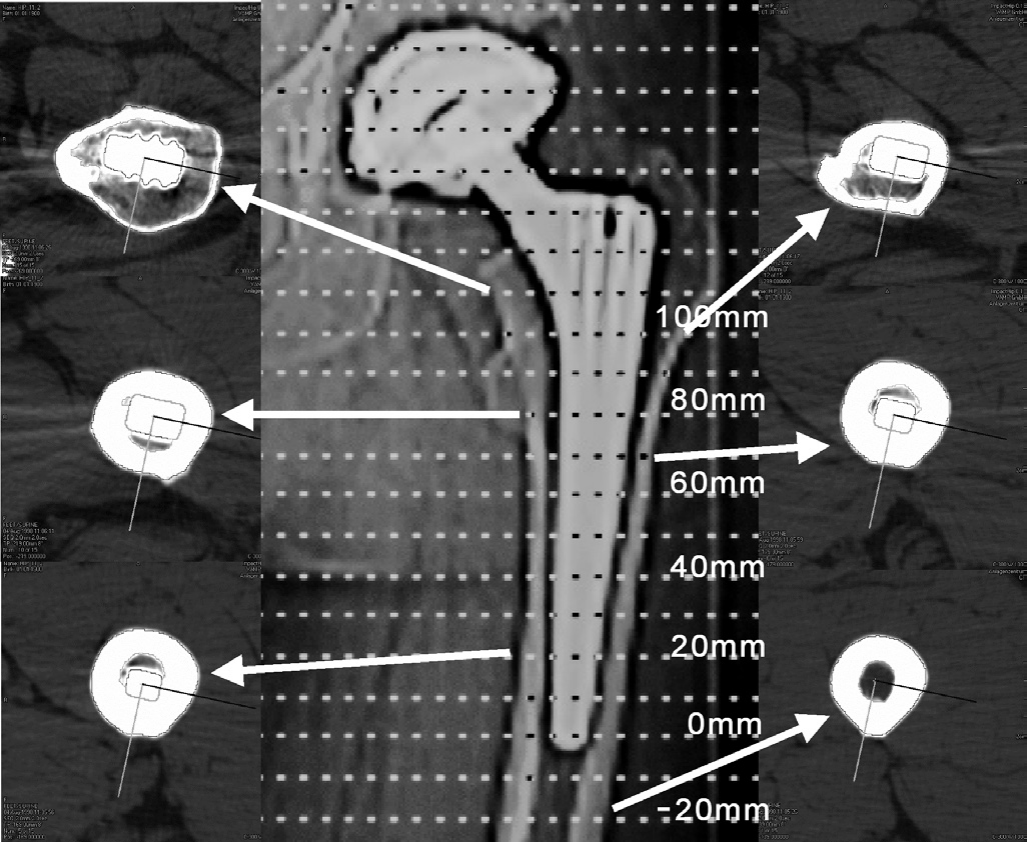
Topogram of an uncemented prosthesis with a selection of CT scans. The first scan of the femur was done 2 cm below the tip of the stem (–20 mm) and the most proximal was done at the 100 mm position.

CT-assisted osteodensitometry (Somatom Plus 4; Siemens, Erlangen, Germany) was performed 10 days, 1 year, and 6 years after the index operation. The study was approved by the local ethics committee. All 31 patients signed an informed consent sheet. A standardized CT protocol and patient positioning was applied (Schmidt et al. 2004). The slide thickness of scans was 2 mm and the table feed (the distance between two scans) was 10 mm. The first scan of the femora was done 2 cm below the tip of the stem (Figure 1). Corresponding measurements were made of the contralateral side. The radiation dose was between 2 and 4 mSv, depending on whether the acetabular component was included in the measurements.

**Figure 2.**
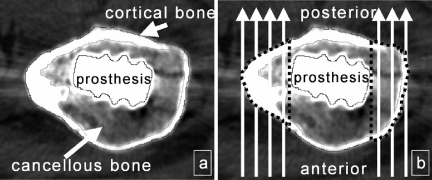
Separate circumferential evaluation of cortical and cancellous BD (mg CaHA/mL) with CT-assisted osteodensitometry (a) compared to the analysis of single zones of interest (dotted lines) in the AP view with DXA (b).

Using a special software tool (CAPPA_postOP; CAS Innovations AG, Erlangen, Germany), the CT scans were automatically evaluated on the basis of an adaptive automatic tracer algorithm that outlined the contours of the outer and inner cortical bone as well as of the prosthesis. Limited manual interaction was required for the correction of inaccurate segmentation, especially due to metal artifacts. Cortical and cancellous BD (mg CaHA/mL) and area (mm^2^), as well as contact area (prosthesis-cortical bone, in %), were analyzed separately (Figure 2) for both the operated side and the unoperated side. Contact area, cortical bone density, and area changes were measured along the entire length of the prosthesis. Due to the extremely low cancellous bone fraction along the diaphysis and the resulting lack of measurement precision, cancellous bone density and area were evaluated in the femoral metaphysis and at the tip of the stem.

### Statistics

The quantitative BD measurements are described as mean values (SD). In order to compare the BD measurements, a mixed linear model with scan position and time as fixed factors and patients as random factor was used. The intra-individual differences between the postoperative measurement and the 1-year and 6-year evaluations were tested post hoc according to the Tukey-Kramer method. All tests were two-sided, and a p-value of less than 0.05 was considered statistically significant. All calculations were done using SAS software version 9.2.

## Results

### Clinical and plain radiograph measurements

None of the femoral or acetabular components required revision surgery. No thigh pain was reported after the index operation. The mean preoperative Harris hip score was rated 58 (41–68) points; at the 6-year follow-up, it improved to 94 (86–97) points, and all patients were rated good or excellent. None of the patients reported any squeaking of the joint. All femoral components were placed in the correct position without valgus or varus deviation. For all femoral stems and cups, there were radiographic signs of stable fixation with complete bony ingrowth.

### CT measurements

The standardized patient positioning and CT-scanning technique allowed an exact evaluation of contact area between cortical bone and the prosthesis, as well as BD and the area of cortical bone, proximal cancellous bone, and cancellous bone at the tip of the stem (relative change in periprosthetic BD as a function of time with the initial postoperative BD value as the baseline value) for all patients regardless of metal artifacts included in the measurements (Tables 1–5). The precision of our measurements was unacceptably low for cancellous BD and area along the femoral diaphysis, with coefficient of variation values of 9.8% and higher for both parameters between scan positions 0 mm and 60 mm.

1 year postoperatively, we found a marked loss of cortical BD at the femoral metaphysis (–11% to –14%). Loss of BD was limited along the diaphysis (–3% to –8%). 6 years postoperatively, cortical BD along the diaphysis and the tip of the stem showed little change; in contrast, we found a progressive decrease in cortical BD at the level of the femoral metaphysis (–16% to –27%) (Table 1).

**Table 1.** Mean cortical bone density measurements from proximal (position 100 mm) to distal (position –20 mm), postoperatively and at follow-up. The coefficient of variation (precision of measurements) is given for each scan position derived from 5 repeated CT scans of 5 cadavers

A	B	C	D	E	F	G	H	I
100 mm	1.2%	891 (77)	770 (103)	–121 (–14%)	< 0.001	649 (92)	–242 (–27%)	< 0.001
90 mm	1.0%	957 (87)	822 (197)	–135 (–14%)	< 0.001	736 (153)	–220 (–23%)	< 0.001
80 mm	0.8%	1,066 (76)	921 (126)	–145 (–14%)	< 0.001	841 (120)	–225 (–21%)	< 0.001
70 mm	0.6%	1,178 (59)	1,049 (104)	–129 (–11%)	< 0.001	989 (106)	–189 (–16%)	< 0.001
60 mm	0.6%	1,210 (74)	1,119 (88)	–92 (–8%)	1.0	1,066 (111)	–144 (–12%)	< 0.001
50 mm	0.9%	1,230 (63)	1,154 (97)	–76 (–6%)	0.1	1,095 (86)	–135 (–11%)	< 0.001
40 mm	0.5%	1,242 (62)	1,161 (90)	–80 (–6%)	0.1	1,120 (79)	–122 (–10%)	< 0.01
30 mm	0.7%	1,245 (61)	1,166 (84)	–79 (–6%)	0.2	1,140 (73)	–106 (–8%)	0.03
20 mm	0.5%	1,250 (61)	1,162 (119)	–87 (–7%)	0.08	1,163 (69)	–87 (–7%)	0.4
10 mm	0.7%	1,249 (70)	1,187 (115)	–61 (–5%)	0.7	1,173 (94)	–76 (–6%)	0.8
0 mm	0.5%	1,246 (80)	1,204 (123)	–42 (–3%)	1.0	1,201 (92)	–45 (–4%)	1.0
–10 mm	0.3%	1,227 (101)	1,170 (130)	–57 (–5%)	0.7	1,179 (87)	–48 (–4%)	1.0
–20 mm	0.2%	1,235 (106)	1,172 (128)	–62 (–5%)	0.4	1,170 (121)	–64 (–5%)	1.0
A Scan position								
B Coefficient of variation								
C Post-operatively **^a^** (mg CaHA/mL)								
D 1 year post-operatively **^a^** (mg CaHA/mL)								
E Difference at 1 year (%)								
F p-value at 1 year								
G 6 years post-operatively **^a^** (mg CaHA/mL)								
H Difference at 6 years (%)								
I p-value at 6 years								
**^a^** values are mean (SD)								

1 year after the operation, we observed a marked decrease in metaphyseal cancellous BD (–16% to –20%). 6 years postoperatively, a progressive loss of cancellous BD was observed in this region (–33% to –41%). 1 year postoperatively, cancellous BD increased at the tip of the stem. At the 6-year follow-up, cancellous BD at the tip of the stem also declined (Table 2).

**Table 2.** Mean cancellous bone density measurements proximally (position 100 mm to 70 mm) and distally (position –10 mm and –20 mm), posoperatively and at follow-up. The coefficient of variation (precision of measurements) is given for each scan position derived from 5 repeated CT scans of 5 cadavers

A	B	C	D	E	F	G	H	I
100 mm	0.9%	124 (60)	104 (63)	–20 (–16%)	0.07	79 (66)	–45 (–36%)	< 0.001
90 mm	0.7%	153 (59)	132 (62)	–21 (–14%)	0.09	91 (64)	–62 (–41%)	< 0.001
80 mm	1.1%	181 (76)	148 (68)	–33 (–18%)	< 0.001	109 (76)	–72 (–40%)	< 0.001
70 mm	1.3%	208 (94)	166 (78)	–42 (–20%)	0.02	139 (82)	–69 (–33%)	< 0.001
–10 mm	0.4%	36 (52)	44 (71)	8 (22%)	0.04	30 (48)	–6 (–17%)	0.06
–20 mm	0.3%	17 (36)	22 (49)	5 (29%)	0.01	14 (20)	–3 (–18%)	0.05
A mild but not statistically significant cortical hypertrophy (2–5%) was seen along the entire length of the diaphysis 1 and 6 years after the index operation (Table 3). Cancellous bone area had increased proximally by up to 19% 1 year postoperatively and by up to 25% 6 years after the index operation (Table 4).								
A–I: See Table 1								

A mild, but not statistically significant, cortical hypertrophy (2–5%) was seen along the entire length of the diaphysis 1 and 6 years after the index operation (Table 3).

**Table 3.** Mean measurements of cortical bone area from proximal (position 100 mm) to distal (position –20 mm), postoperatively and at follow-up. The coefficient of variation (precision of measurements) is given for each scan position derived from 5 repeated CT scans of 5 cadavers

A	B	C	D	E	F	G	H	I
100 mm	2.3%	1,300 (264)	1,293 (184)	–7 (–1%)	1.0	1,414 (229)	114 (9%)	0.02
90 mm	1.5%	1,055 (212)	1,078 (187)	23 (2%)	1.0	1,175 (193)	120 (11%)	0.2
80 mm	2.5%	862 (143)	891 (149)	29 (3%)	1.0	922 (157)	60 (7%)	1.0
70 mm	0.9%	741 (107)	773 (114)	32 (4%)	1.0	786 (125)	46 (6%)	1.0
60 mm	0.8%	694 (98)	714 (99)	20 (3%)	1.0	731 (116)	37 (5%)	1.0
50 mm	0.4%	665 (94)	684 (96)	19 (3%)	1.0	696 (105)	31 (5%)	1.0
40 mm	0.7%	650 (95)	663 (94)	13 (2%)	1.0	675 (102)	25 (4%)	1.0
30 mm	0.6%	642 (97)	654 (96)	11 (2%)	1.0	661 (101)	19 (3%)	1.0
20 mm	0.6%	637 (100)	655 (107)	18 (3%)	1.0	654 (102)	17 (3%)	1.0
10 mm	0.4%	635 (109)	657 (126)	22 (3%)	1.0	660 (121)	25 (4%)	1.0
0 mm	0.4%	636 (122)	662 (147)	26 (4%)	1.0	662 (141)	26 (4%)	1.0
–10 mm	0.1%	643 (143)	665 (174)	21 (3%)	1.0	663 (161)	19 (3%)	1.0
–20 mm	0.1%	650 (176)	668 (203)	18 (3%)	1.0	665 (192)	15 (2%)	1.0
A–I: See Table 1.								

Cancellous bone area increased proximally by up to 19% 1 year postoperatively and by up to 25% 6 years after the index operation (Table 4).

**Table 4.** Mean measurements of cancellous bone area proximally (position 100 mm to 70 mm) and distally (position –10 mm to –20 mm), postoperatively and and at follow-up. The coefficient of variation (precision of measurements) is given for each scan position deriving from five repeated CT-scans of 5 cadavers

A	B	C	D	E	F	G	H	I
100 mm	1.4%	910 (194)	967 (168)	56 (6%)	0.2	1,055 (174)	144 (16%)	< 0.01
90 mm	1.1%	674 (162)	739 (160)	66 (10%)	0.8	840 (153)	166 (25%)	< 0.001
80 mm	0.5%	480 (112)	567 (135)	87 (18%)	0.07	599 (119)	118 (25%)	< 0.001
70 mm	0.4%	354 (74)	421 (78)	67 (19%)	0.05	440 (100)	87 (25%)	< 0.001
–10 mm	0.3%	191 (105)	197 (106)	7 (4%)	0.7	207 (157)	17 (9%)	1.0
–20 mm	0.4%	201 (137)	219 (160)	18 (9%)	0.9	207 (132)	7 (3%)	1.0
A–I: See Table 1.								

On the unoperated side, no significant changes in area and BD were observed for cortical and cancellous BD.

The contact area between the prosthesis and the inner cortical bone was highest between scan positions 30 mm and 60 mm (with up to 27% contact area at scan position 50 mm one year postoperatively). Changes in contact area were not significant 1 and 6 years postoperatively (Table 5).

**Table 5.** Contact area measurements (% out of a possible 100% contact area between the prosthesis and the inner cortical bone) from proximal (position 100 mm) to distal (position 0 mm), postoperatively and at follow-up. The coefficient of variation (precision of measurements) is given for each scan position derived from 5 repeated CT scans of 5 cadavers

A	B	C	D	E	F	G	H	I
100 mm	3.8%	0.08 (0.08)	0.09 (0.08)	0.02	1.0	0.08 (0.06)	0.00	1.0
90 mm	4.4%	0.10 (0.09)	0.15 (0.23)	0.05	1.0	0.08 (0.06)	–0.02	1.0
80 mm	3.4%	0.11 (0.09)	0.10 (0.08)	–0.01	1.0	0.08 (0.06)	–0.04	1.0
70 mm	4.1%	0.13 (0.11)	0.16 (0.07)	0.04	1.0	0.12 (0.08)	–0.01	1.0
60 mm	2.7%	0.18 (0.10)	0.25 (0.14)	0.07	1.0	0.15 (0.13)	–0.03	0.4
50 mm	2.2%	0.17 (0.14)	0.27 (0.16)	0.09	1.0	0.14 (0.12)	–0.03	1.0
40 mm	1.8%	0.18 (0.15)	0.21 (0.13)	0.03	1.0	0.16 (0.12)	–0.03	1.0
30 mm	2.9%	0.18 (0.12)	0.19 (0.12)	0.01	1.0	0.14 (0.10)	–0.04	1.0
20 mm	2.7%	0.17 (0.13)	0.17 (0.10)	0.00	1.0	0.15 (0.11)	–0.02	1.0
10 mm	3.1%	0.14 (0.09)	0.24 (0.13)	0.10	0.9	0.21 (0.14)	0.07	1.0
0 mm	1.5%	0.10 (0.10)	0.28 (0.15)	0.18	0.2	0.28 (0.15)	0.18	0.09
A–I: See Table 1.								

## Discussion

### Clinical and plain radiographic measurements

The outcome of uncemented THA depends on many factors, including patient selection, bone stock at index operation, component design, and surgical technique. In accordance with the results reported by other authors using taper-design stems (Johnston et al. 1990, Schramm et al. 2000, Brodner et al. 2004), our clinical and radiographic results at a mean of 6 years were satisfactory.

### CT measurements

Longitudinal DXA studies have revealed a limited restoration of periprosthetic BD between the first and the second to third postoperative year (Nishii et al. 1997, Rosenthall et al. 1999, Venesmaa et al. 2001). They suggest that the bone response in the second and third postoperative year might be indicative of good osteointegration, reflected by good load transfer (Venesmaa et al. 2001) and return of the patients to normal physical activity (Kannus et al. 1994, Rosenthall et al. 1999, Venesmaa et al. 2001). We found continuous decrease in proximal cortical BD between the first (Ø–13%) and sixth (Ø–22%) postoperative year. We observed limited, non-progressive decrease in cortical BD at the femoral diaphysis (–6% and –9%, respectively, one and 6 years postoperatively) and at the tip of the stem (–4% and –4% one and 6 years postoperatively). All our patients had returned to normal physical activity with excellent to good clinical results. We suggest that continuous stress shielding was responsible for the proximal cortical BD loss observed between the first and sixth postoperative year while a homeostatic cortical strain configuration had been achieved at the femoral diaphysis 6 years after the index operation. The rationale of a metaphyseal fixation of the Multicone uncemented, tapered femoral component seems not to be confirmed in these clinical settings.

With our methodology, proximal cancellous BD was measured for the first time in vivo. Cancellous BD changes below the tip of the stem (positions –10 mm and –20 mm) should be interpreted carefully, as we found very low absolute values (13 to 36 mg CaHA/mL) at all time points. Differences between the baseline BD values and the BD values at 1- and 6-year follow-up were small (–6 to +9 mg CaHA/mL). We assume that these minor changes are within the range of accuracy of our densitometry system.

The proximal cancellous BD loss measured was high and progressive (Ø –18% 1 year after surgery, Ø –38% 6 years after surgery). These results can be interpreted in two ways: either as a lack of proximal mechanical loading as suggested for cortical bone or as a stress-induced cancellous bone failure (Taylor et al. 1995a, b). Wolff's law describes the biological reaction pattern to mechanical stimuli, but it is not a law in a physical sense. Mechanical loading, which might induce a gain in cortical BD, could theoretically exceed the strength of cancellous bone, disturb the equilibrium of cancellous bone damage and healing, and thus lead to its collapse (Taylor et al. 1995a, b).

The in vivo measurement of periprosthetic cancellous bone density by CT-assisted ostedensitometry as described in our study may give us further insights in our understanding of aseptic loosening, and predict stem failure at the early stages. Some authors have suggested compaction or resorption of periprosthetic bone (Linder 1994) to be responsible for medium-term migration, the rate of which has been linked to later failure (Mjöberg et al. 1986). The prosthesis is supported by cancellous bone in the proximal region especially; medium-term migration may thus be due to failure of cancellous bone caused by excessive mechanical stress (Taylor and Tanner 1997). Finite-element studies have shown increased stress in the cancellous bone that may exceed the in vitro fatigue stress of such bone, resulting in the collapse of cancellous bone over an extended period (Taylor et al. 1995a, b). The extensive loss in proximal cancellous BD observed 6 years after the operation may contribute to the process of aseptic loosening by creating an area of poor-quality cancellous bone that is vulnerable to ultra-high molecular weight polyethylene (Santavirta et al. 1990).

Further long-term investigations, possibly in combination with migration analysis, may show whether patients with a high degree of cancellous BD loss have a higher risk of medium-term migration and whether they are more susceptible to development of osteolytic bone lesions. Both migration and osteolytic lesions reduce the longevity of implants.

Bobyn et al. (1992) observed a 25–35% increase in cortical area on average in histologically analyzed femora of dogs that had received a titanium-alloy THA 1 year postoperatively. Such high increases in cortical area seem unlikely in humans after cementless, tapered titanium alloy THA as shown in our study (2–5% increase in cortical area). There are no published CT data on the development of cortical and cancellous areas after canal-filling THA, which are known to show high rates of distal cortical hypertrophy on plain radiographs (Engh and Culpepper 1997, Aldinger et al. 2003, Grübl et al. 2006).

Pure metaphyseal load transfer, as hypothesized by the designers of similar tapered stems (Spotorno et al. 1993), cannot be achieved with the press-fit femoral component analyzed here. Progressive gain in cancellous bone area (up to 25% 6 years postoperatively), mild cortical hypertrophy (2–5%) along the entire length of the diaphysis, and the highest level of contact area between the prosthesis and the inner cortical bone between scan positions 30 mm and 60 mm all suggest diaphyseal loading of the prosthesis.

To summarize, the clinical relevance of progressive loss of metaphyseal cancellous BD and gain in cancellous area and their relationship with mechanical loosening of the implant remains unclear. Even so, separate analysis of cortical and cancellous BD and area with CT-assisted osteodensitometry may be predictive of stem failure at an early stage, and may possibly detect osteolytic lesions long before they are visible on plain radiographs. CT-assisted osteodensitometry has the potential to become an effective instrument for quality control in total hip arthroplasty, enabling the development of screening procedures before new prostheses are introduced to the general public. Future studies should further elucidate the role of progressive loss of proximal cancellous BD in migration and in generating osteolytic lesions.
